# Image-based AI diagnostic performance for fatty liver: a systematic review and meta-analysis

**DOI:** 10.1186/s12880-023-01172-6

**Published:** 2023-12-11

**Authors:** Qi Zhao, Yadi Lan, Xunjun Yin, Kai Wang

**Affiliations:** 1 Department of Gastroenterology, Shandong Provincial Hospital, Shandong University, Jinan, Shandong 250021 China; 2 Department of Hepatology, Institute of Hepatology, Qilu Hospital of Shandong University, Shandong University, Wenhuaxi Road 107#, Jinan, Shandong 250012 China; 3 Department of Gastroenterology, Shandong Provincial Hospital Affiliated to Shandong First Medical University, No. 324 Jingwu Road, Jinan, Shandong 250021 China; 4 Shandong Booke Biotechnology Co. LTD, Liaocheng, Shandong China

**Keywords:** Artificial intelligence, Imaging, Diagnosis, Fatty liver, Meta-analysis

## Abstract

**Background:**

The gold standard to diagnose fatty liver is pathology. Recently, image-based artificial intelligence (AI) has been found to have high diagnostic performance. We systematically reviewed studies of image-based AI in the diagnosis of fatty liver.

**Methods:**

We searched the Cochrane Library, Pubmed, Embase and assessed the quality of included studies by QUADAS-AI. The pooled sensitivity, specificity, negative likelihood ratio (NLR), positive likelihood ratio (PLR), and diagnostic odds ratio (DOR) were calculated using a random effects model. Summary receiver operating characteristic curves (SROC) were generated to identify the diagnostic accuracy of AI models.

**Results:**

15 studies were selected in our meta-analysis. Pooled sensitivity and specificity were 92% (95% CI: 90–93%) and 94% (95% CI: 93–96%), PLR and NLR were 12.67 (95% CI: 7.65–20.98) and 0.09 (95% CI: 0.06–0.13), DOR was 182.36 (95% CI: 94.85-350.61). After subgroup analysis by AI algorithm (conventional machine learning/deep learning), region, reference (US, MRI or pathology), imaging techniques (MRI or US) and transfer learning, the model also demonstrated acceptable diagnostic efficacy.

**Conclusion:**

AI has satisfactory performance in the diagnosis of fatty liver by medical imaging. The integration of AI into imaging devices may produce effective diagnostic tools, but more high-quality studies are needed for further evaluation.

**Supplementary Information:**

The online version contains supplementary material available at 10.1186/s12880-023-01172-6.

## Background

Fatty liver disease has become more and more prevalent in recent years [1], making it the most common chronic liver disease in the world. Fatty liver can lead to steatohepatitis, liver fibrosis, cirrhosis, and even hepatocellular carcinoma, early detection and treatment may stop or even reverse the progression of fatty liver [2]. The best reference for diagnosis and classification of hepatic steatosis is the liver biopsy [3]. Nevertheless, the high cost [4], sampling errors [5, 6], and procedure-related morbidity and mortality [7] make it unsuitable for screening. Therefore, it is urgent and necessary to develop non-invasive diagnostic tools to assess hepatic steatosis.

Imaging is a useful tool to assist decisions of diagnosis, staging, and treatment in clinical practice. Currently, the main diagnostic modalities by medical imaging for fatty liver include magnetic resonance imaging (MRI), ultrasound (US), and computed tomography (CT). Conventional US is cheap, safe, and non-invasive, so it is the most commonly used modality for clinical screening [8]. But the diagnostic accuracy in the US is largely dependent on personal judgment which may be susceptible to many factors. CT can effectively detect fatty liver without the influence of abdominal fat. But it is radioactive and expensive, besides, the classification of fatty liver by CT value may be too rough. MRI has high soft tissue resolution and can quantify intrahepatic fat at the molecular level, so it is the main modality for the non-invasive quantification of hepatic steatosis [9]. However, the high cost and difficult operation may limit its clinical application. In institutions with limited medical resources, the lack of imaging equipment and experts will make it challenging to obtain the accurate and immediate diagnosis through medical imaging [10].

Artificial intelligence(AI) has made significant advances since the 21st century, especially in medical imaging diagnosis [11], such as conventional machine learning(ML) and deep learning(DL). Concerning the application of AI in medical imaging, a large number of quantitative features can be extracted from radiological images using sophisticated image processing techniques, which are subsequently analyzed by traditional biostatistical or AI models to diagnose or assess therapeutic responses. Several AI-assisted diagnostic models have been developed for fatty liver, such as Han et al. [12] who developed a classifier for the diagnosis of nonalcoholic fatty liver disease(NAFLD), obtaining 97% for sensitivity and 94% for specificity. The model was established by DL using US radio frequency (RF) data with reference to MRI-derived proton density fat fraction (PDFF). Many scholars are trying to improve the diagnostic efficacy of AI models by optimizing image quality, expanding sample size, and modifying algorithms.

To date, little meta-analysis has been conducted to evaluate the diagnostic performance of image-based AI. The study aimed to perform a systematic review and meta-analysis to assess the performance of image-based AI in the diagnosis of fatty liver.

## Methods

### Protocol registration and study design

The study was registered in the PROSPERO(CRD42023388607). The meta-analysis took the Preferred Reporting Items for Systematic Review and Meta-Analysis (PRISMA) guideline [13] as the reference.

### Search strategy

We searched Embase, Pubmed, and Cochrane library for studies of image-based AI in fatty liver until December 24, 2022. The search terms were as follows: “artificial intelligence”, “deep learning”, “machine learning”, “fatty liver”, “NAFLD”, “non-alcoholic fatty liver disease”, “steatohepatitis”, “metabolic dysfunction-associated fatty liver disease” and “diagnosis, computer-assisted”. The detailed search strategies for each database were summarised in Table S1.

### Inclusion and exclusion criteria

We included all articles that used AI in the imaging diagnosis of hepatic steatosis. The inclusion criteria: (1) participants underwent fatty liver-related imaging; (2) references were accurately described. The exclusion criteria: (1) duplicate publications; (2) non-English articles; (3) reviews, meta-analyses, comments, editorials, guidelines, and conference abstracts; (4) non-human samples; (5) pathological images, combined with non-image information, without AI models; (6) studies without enough information to calculate true positive (TP), true negative (TN), false positive (FP), and false negative (FN) values. The titles and abstracts were independently screened according to the eligibility criteria by two reviewers (L-YD and Z-Q), and subsequently downloaded and reviewed the full text.

### Data extraction

Two authors (L-YD and Z-Q) conducted the data extraction independently. Any disagreements about the data were determined with the third author(Y-XJ). Data extraction included authors, years, countries, study design, eligibility criteria, age, sample size, data source and range, imaging technique, reference, AI algorithm, and TP, FP, TN, FN values, which were used to calculate sensitivity and specificity. For studies that developed more than one AI model, we selected the one with the best overall performance for analysis.

### Quality assessment

Two independent evaluators (L-YD and Z-Q) assessed the quality of all selected studies by the Quality Assessment of Diagnostic Accuracy Studies-AI (QUADAS-AI) criteria [14]. The guideline includes four domains in the risk of bias and three domains of applicability (Table S2). The new tool, a combination of QUADAS-2 [15] and QUADAS-C [16], was specifically designed to assess the risk and suitability of bias in AI associated studies. All disagreements were discussed with a third collaborator (Y-XJ).

### Statistical analysis

The quality of all selected studies was assessed by RevMan using QUADAS-AI, the risk of publication bias was assessed by Stata software (version 17.0) and all other statistical analysis was conducted in Meta-disc (version 1.4). Spearman’s correlation coefficient between the log of sensitivity and the log of (1-specificity) was calculated to test the threshold effects, and heterogeneity was tested using the I^2^ statistic. A random effects model was used to calculate pooled sensitivities, specificities, negative likelihood ratios (NLR), positive likelihood ratios (PLR), diagnostic odds ratios (DOR), and their 95% confidence intervals(CI) based on crude values of TP, TN, FP and FN values for each study. Summary receiver operating characteristic curves (SROC) were fitted to assess the accuracy of the AI models. The low, medium, and high accuracy were defined as the area under the curve (AUC) values of 0.5–0.7, 0.7–0.9, and 0.9-1 respectively [17]. Subgroup analyses were then performed: (1) AI algorithm (conventional ML or DL); (2) region; (3) whether transfer learning was performed; (4) reference (US, MRI or pathology); (5)imaging techniques (conventional US, elastography or MRI). The risk of publication bias was assessed by Deeks funnel plots. Fagan plots were drawn to calculate the pre-test and post-test probabilities to evaluate the clinical value. P-values < 0.05 were then considered statistically significant.

## Results

### Study selection

**Fig. 1:**
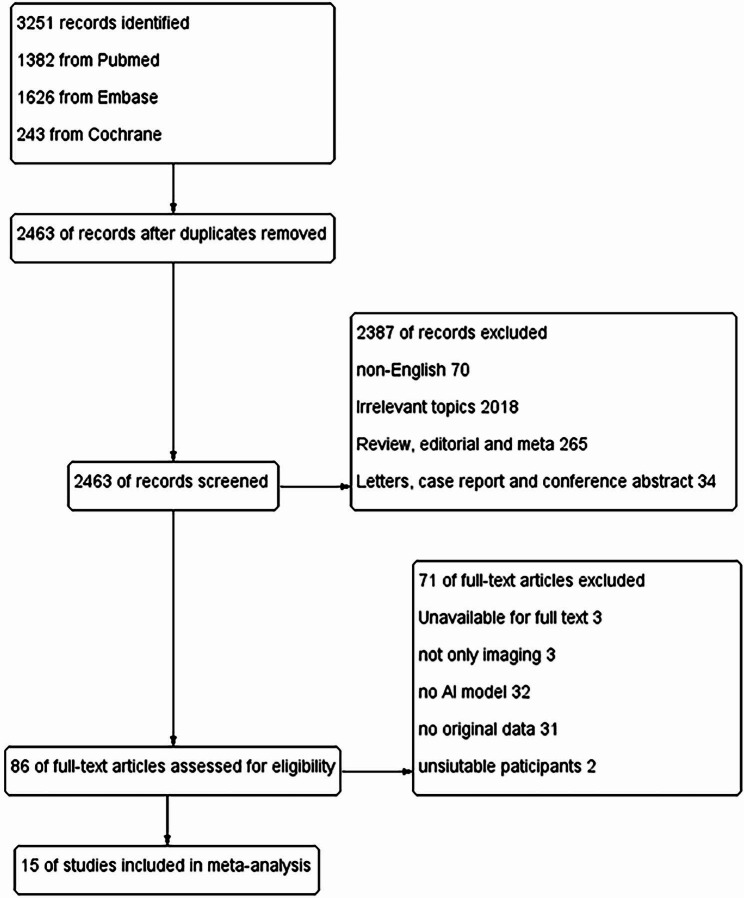
The flow of searching and selecting articles

The flow of searching and selecting articles was shown in Fig. 1. Finally, 15 articles [12, 18–31] were taken into the quantitative analysis. The description of all selected studies was presented in Table 1.

**Table 1:** Characteristic of studies

Author	Year	study design	country	Single/Multi center	Inclusion criteria	Exclusion criteria	age	Source of data	Data range(time)	imaging technique	Reference	Algorithm
G.Li et al.	2008	Retrospective	China	Single	NR	NR	NR	WuHan municipal hospital	NR	Conventional ultrasound	US	SVM
M.Hájek et al.	2011	Retrospective	Czech Republic	Single	With transplanted livers for 1–11 years who underwent regular clinical examination, including protocol liver biopsies.	NR	NR	NR	March 2009 to November 2009 and March 2010 to July 2010	MR spectrum	pathology	Linear regression
U.R.Acharya et al.	2012	Retrospective	Singapore	Single	NR	NR	NR	NR	NR	Conventional ultrasound	US	Decision Tree
F.U.A.A.Minhas et al.	2012	Retrospective	USA	Single	NR	NR	NR	Multan Institute of Nuclear Medicine and Radiotherapy (MINAR)	NR	Conventional ultrasound	US	SVM
R.Ribeiro et al.	2012	Retrospective	Portugal	Single	had known diagnosis based on liver biopsy results	NR	NR	Hospital de Santa Maria, Medical School of Lisbon, Portugal	NR	Conventional ultrasound	pathology	Bayes
R.T.Ribeiro et al.	2014	Retrospective	Portugal	Single	All patients were outpatients with known diagnosis, based on the clinical and US criteria.	NR	NR	The Gastroenterology Department of the Santa Maria Hospital, Lisbon	NR	Conventional ultrasound	US	Bayes
M.Owjimehr et al.	2015	Retrospective	Iran	NR	NR	NR	NR	NR	NR	Conventional ultrasound	US	SVM
L.Saba et al.	2016	Retrospective	Italy	Single	NR	NR	NR	NR	NR	Conventional ultrasound	US	BPNN
V.Kuppili et al.	2017	Retrospective	India	Single	The patient with normal body mass index.	NR	NR	Instituto Superior Tecnico (IST), University of Lisbon, Portugal	NR	Conventional ultrasound	pathology	ELM
M.Biswas et al.	2018	Retrospective	India	Single	NR	NR	NR	The Santa Maria Hospital (ethics approval granted), in Lisbon, Portugal	NR	Conventional ultrasound	pathology	CNN
V.Sharma et al.	2018	Retrospective	India	Single	age group of 25–60 years old	NR	NR	Delta Diagnostic Centre Patiala, India	NR	Conventional ultrasound	US	SVM
A.Han et al.	2020	Prospective	Poland	Single	Age of at least 18 years, willingness and ability to participate in.	Clinical, laboratory, or histologic evidence of a liver disease other than NAFLD; excessive alcohol consumption (0.30 g per day within the past 10 years or 0.10 g per day in the previous year); and steatogenic or hepatoxic medication use.	NAFLD 52 ± 14; control 46 ± 21	The University of California, San Diego, NAFLD Research Center	Between February 2012 and March 2014	Conventional ultrasound	MRI	CNN
E.C.Constantinescu et al.	2021	Retrospective	Romania	Single	Age ranging from 18 to 92 from the outpatient clinic of a private healthcare network.	Excessive alcohol consumption or > 20 g ethanol per day, history or clinical and/or laboratory evidence of liver disease, hepatotoxic medication use.	NR	The University of Medicine and Pharmacy, Research Center of Gastroenterology and Hepatology, Craiova from the outpatient clinic of a private healthcare network.	NR	B-mode ultrasound and ARFI elastography	US	Inception v3
M.Byra et al.	2022	Prospective	USA	Single	Age ≥ 18 years old, known or suspected NAFLD, and willingness and ability to participate.	Clinical, laboratory, or histology evidence of a liver disease other than NAFLD, excessive alcohol consumption (≥ 14 (men) or ≥ 7(women)drinks/week), and steatogenic or hepatoxic medication use.	NR	The University of California, San Diego (UCSD)	Between 2016 and 2018	Conventional ultrasound	MRI	ResNet50 CNN;LR
F.Destrempes et al.	2022	Retrospective	Canada	Single	A liver biopsy was scheduled as part of their clinical standard of care.	(a) images were not acquired with the probe assigned to the study protocol; (b) the underlying pathology did not meet eligibility criteria due to ethanol consumption, drug-induced hepatitis, sarcoidosis, cholestasis, or no biopsy was performed.		Centre hospitalier de l’Universite´ de Montreál and McGill University Health Centre	Between October 2014 and September 2018	Quantitative ultrasound(QUS) and Point shear wave elastography (pSWE)	pathology	Random forest

Abbreviations: NR: not report; USA: United States of America; US: ultrasound; MRI: magnetic resonance imaging; BPNN: Back Propagation Neural Networks; CNN: Convolutional Neural Network; SVM: Support Vector Machine; ELM: extreme learning machine; ARFI: acoustic radiation force impulse

### Quality assessment

The detailed results of quality assessments of included studies were presented in Figure S1. The risk of bias was shown in more than half of the studies for patient selection (n = 8) and index test (n = 15) because of the lack of detailed descriptions of included patients and appropriate external validation.

### Overall performance of AI models

**Fig. 2:**
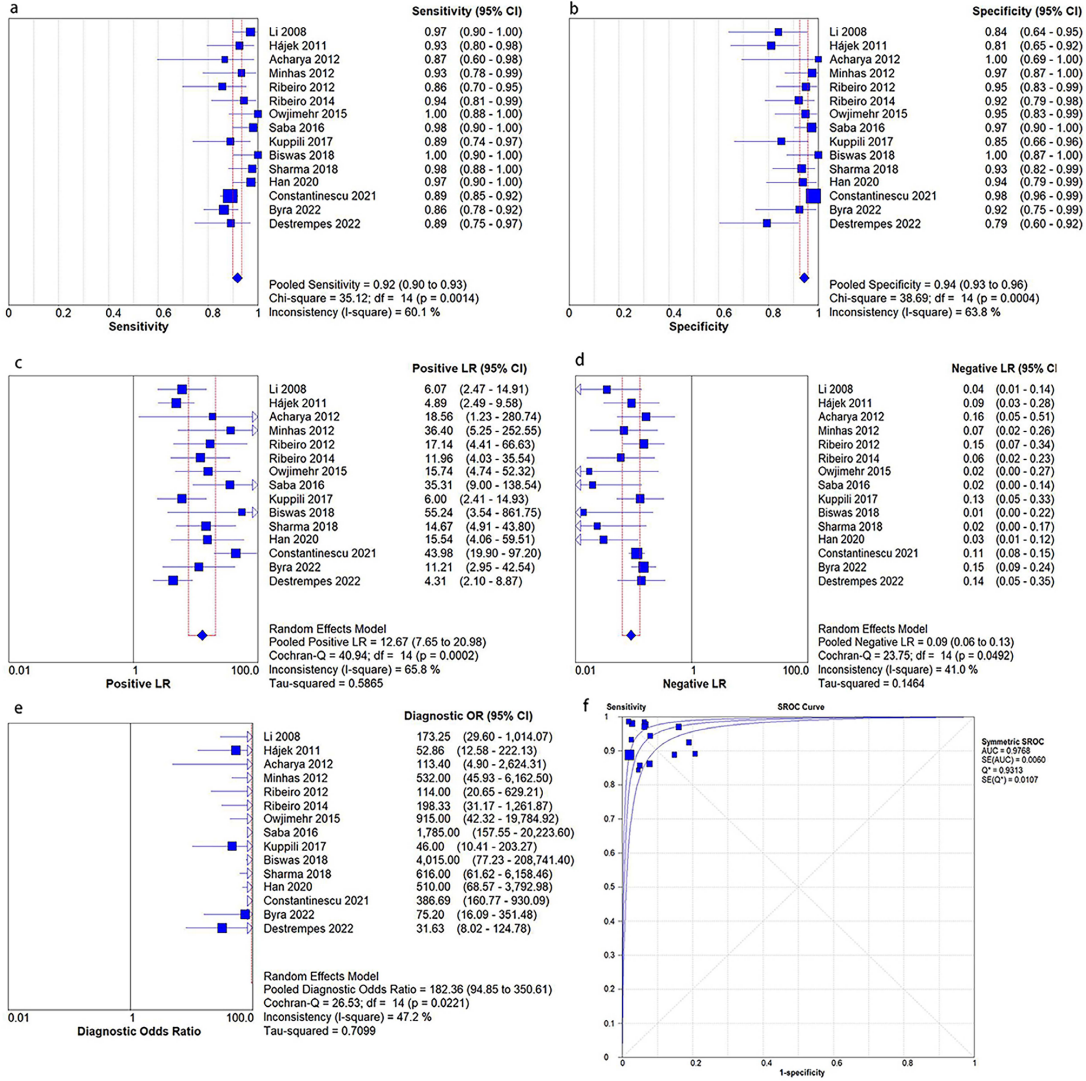
Forest plot of all AI models. (**a**) The pooled sensitivity; (**b**) The pooled specificity; (**c**) The pooled positive likelihood ratio (PLR); (**d**) The pooled negative likelihood ratio (NLR); (**e**) The pooled diagnostic odds ratio (DOR); (**f**) The summary receiver operating characteristic curves (SROC)

The detailed information on contingency tables and performance of AI models from 15 included studies was shown in Table S3. The meta-analysis indicated that image-based AI models were effective to diagnose liver steatosis with pooled sensitivity and specificity of 92% (95% CI: 90–93%) and 94% (95% CI: 93–96%), PLR and NLR of 12.67 (95% CI: 7.65–20.98) and 0.09 (95% CI: 0.06–0.13), DOR of 182.36 (95% CI: 94.85-350.61), and SROC of 0.98. (Fig. 2).

### Subgroup meta-analysis

We performed the subgroup analysis of AI algorithm, region, reference, imaging technique and transfer learning. In AI algorithm, the pooled sensitivity and specificity of 9 conventional ML studies were 94% (95% CI: 91–96%) and 91% (95% CI: 87–94%), and were 91% (95% CI: 88–93%) and 97% (95% CI: 95–98%) in 6 DL studies. For different regions, 6 studies were conducted in Asia with pooled sensitivity and specificity of 96% (95% CI: 93–98%) and 92% (95% CI: 87–96%), 9 studies were in Europe and America with pooled sensitivity and specificity of 90% (95% CI: 88–92%) and 95% (95% CI: 93–97%). For different references, the sensitivities of US, pathology and MRI were 92%, 91% and 92%, and the specificities were 97%, 88%, and 90% respectively. For different imaging techniques, the sensitivities of conventional US, elastography and MRI were 94%, 89% and 93%, and the specificities were 94%, 96%, and 81%. Two studies employed transfer learning with pooled sensitivity and specificity of 88% (95% CI: 85–91%) and 98% (95% CI: 97–99%), 13 studies did not perform transfer learning with pooled sensitivity and specificity of 95% (95% CI: 92–96%) and 92% (95% CI: 89–94%). The details of subgroup analysis were shown in Table S4.

### Heterogeneity analysis

There was substantial heterogeneity between the included studies, with I^2^ = 60.1% (p = 0.001) for sensitivity, I^2^ = 63.8% (p < 0.001) for specificity, I^2^ = 65.8% (p < 0.001) for PLR, I^2^ = 41.0% (p = 0.049) for NLR, I^2^ = 47.2% (p = 0.022) for DOR. The Spearman correlation coefficient was − 0.148 (p = 0.598), indicating that there was no threshold effect. And the heterogeneity was reduced after subgroup analysis which were presented in Table S4.

### Clinical value and publication bias

**Fig. 3:**
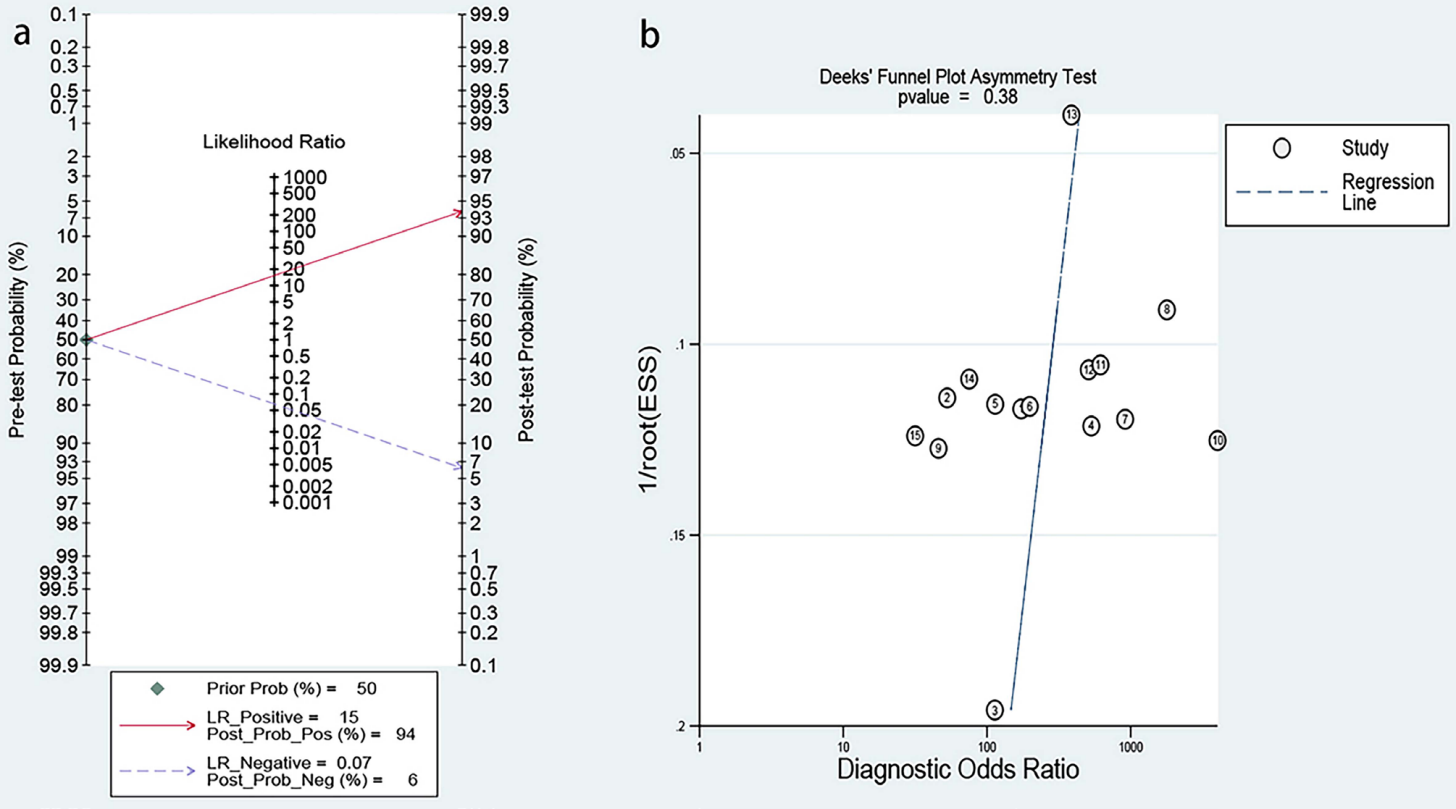
Clinical value and publication bias: (**a**) Fagan plot; (**b**) Deeks’ funnel plot

The post-test probability of image-based AI for the diagnosis of hepatic steatosis was 94%, much higher than the pre-test probability (50%), indicating that image-based AI is valid for the diagnosis of hepatic steatosis (Fig. 3a). And the Deeks funnel plot revealed no obvious publication bias of included studies (P = 0.38) (Fig. 3b).

## Discussion

AI has been widely used in medical imaging in recent years, so more and more AI models have been established to diagnose various liver diseases [32, 33]. We conducted an extensive literature search in medical databases, which was carefully screened and critically assessed by QUADAS-AI. Ultimately, we found that AI models performed well in identifying liver steatosis by medical imaging.

AI aims to simulate, extend and expand human intelligence [34]. Conventional ML is the method to achieve AI, which can use features extracted from various kinds of data to build prediction models through different algorithms. However, it requires manual extraction of features [35] and the ability of conventional ML to learn from the data was limited [36]. DL is the advanced classification of conventional ML which can utilize multiple layers of deep neural networks for a deeper understanding of the data [37]. However, DL is prone to overfitting and usually requires more data [38]. Our subgroup analysis of the different algorithms showed that the sensitivity was higher in conventional ML, but the specificity, PLR, DOR and SROC were higher in DL. The results revealed the potential advantages of DL in the image-based diagnosis of liver steatosis.

Machine learning is commonly employed in biomedical fields. However, due to insufficient labeled data, the application of advanced machine learning algorithms in clinical settings is limited. Collecting labeled data is time-consuming, energy-draining, and requires professional expertise. To address this problem, transfer learning can transfer the acquired knowledge and models from one related task to another, leading to enhanced performance and generalization of the target task [39]. For instance, a recent study utilized transfer learning to diagnose corona virus disease (COVID-19) automatically through CT images with a remarkable accuracy of 99.60% [40]. In our subgroup analysis, we found that transfer learning led to higher specificity, PLR, and DOR, which highlighted the significance of transfer learning in image-based AI diagnosis of hepatic steatosis. However, only two studies exploited transfer learning, further studies are needed to confirm its effectiveness.

The gold standard for the diagnosis of hepatic steatosis is pathology, but there are diagnostic errors in the liver biopsy due to the limitation of sampling. The EASL Clinical Practice Guideline [41] demonstrated that the MRI-PDFF was the most accurate non-invasive method for detecting and quantifying steatosis. So the articles which used experts diagnostic US or MRI-PDFF as references were also selected in our study. We further conducted the subgroup analysis of different reference standards. The results showed a higher sensitivity and lower specificity in studies taking pathology as the reference compared to US and MRI. This result indirectly demonstrated the previously mentioned limitations of pathology in terms of sampling error. Only part of the liver tissues was taken for pathological examination. When steatosis was slight or focal, false negatives were likely to occur, resulting in low specificity of AI model diagnosis. Therefore, there is an urgent need for image-based AI models with high diagnostic efficacy, which can be integrated into imaging examination equipment.

For the imaging technique, conventional US, elastography and MRI were included in the selected citations. The subgroup analysis of different imaging techniques showed that the sensitivity and DOR was higher in conventional US than elastography and MRI, which demonstrated that AI seems to be more useful in the conventional US. However, the researches on elastography and MRI were too limited, and the source of the data were different. In the future, further researches are needed to explore the AI-assistant efficacy of different imaging techniques.

Additionally, in our subgroup analysis of different regions, we found the sensitivity, DOR and SROC were higher in Asia, the specificity and PLR were higher in Europe and America, which suggested that the regions of included population may influence the diagnostic efficacy of AI for the diagnosis of hepatic steatosis. Most of the studies we included were based on US images, which are susceptible to body size and visceral fat [42]. Westerners are fatter than Asians with greater differences between populations, which may affect the accuracy of AI diagnosis. In the future, an accurate description such as body size and visceral fat of included populations will be needed, so that we can explore the potential influences on the diagnostic efficacy of AI for hepatic steatosis.

There are some advantages in our study. Our study shows the high efficacy of image-based AI in diagnosing hepatic steatosis without publication bias and may provide a reference for future clinical practice. Compared with the previous systematic review of AI-assistant in NAFLD [43], our study mainly explore the diagnostic performance of image-based AI for liver steatosis rather than fibrosis. The number of cited papers (15 citations) was increased and the subgroup analysis of different imaging techniques, AI algorithm, regions and so on may be helpful for future researches. In addition, we employed the new tool QUADAS-AI involve in AI-specific methodology in our study. In the past, the most frequently utilized quality assessment tool for the diagnostic meta-analysis was QUADAS-2. However, it does not involve in AI-specific methodology, such as generalizability and diversity in patient selection, development of training, validation and testing datasets, as well as definition and evaluation of an appropriate reference standard [44]. This new tool QUADAS-AI^14^ is an AI-specific extension of QUADAS-2 and QUADAS-C, includes four domains in the risk of bias and three domains in applicability concerns, which is more comprehensive and suitable for AI associated studies. Some studies [45–47] related to AI models have also employed this new tool.

However, our study has some limitations: firstly, most of the studies were retrospective and did not clearly describe the participants, making it difficult to control many confounding factors. Secondly, none of the included studies underwent suitable external validation, so whether the model can be applied to other populations requires further validation. Finally, there was heterogeneity in our meta-analysis, but no significant threshold effects were found according to Spearman’s correlation coefficient and the heterogeneity was reduced in the subgroups which might be the potential resources of the heterogeneity. In the future, we hope that more prospective AI studies with external validation based on large sample sizes can accurately assess the performance of image-based AI in diagnosing liver steatosis.

## Conclusion

This meta-analysis suggested that AI had vast potential for image-based diagnosis of hepatic steatosis. The integration of AI into imaging devices may produce effective diagnostic tools, but more high-quality studies are needed for sufficient validation.

### Electronic supplementary material

Below is the link to the electronic supplementary material.

**Supplementary Material 1: Table S1.** Search strategies; **Table S2.** Description of QUADAS-AI; **Table S3.** Performance of models from 15 included studies; **Table S4.** Pooled effects and Heterogeneity in subgroup analysis; **Figure S1.** Quality assessment of included articles using the QUADAS-AI criteria.

## Data Availability

Not applicable.

## Abbreviations

AI: Artificial intelligence
NAFLD: Non-alcoholic fatty liver disease
NLR: Negative likelihood ratio
PLR: Positive likelihood ratio
DOR: Diagnostic odds ratio
CI: Confidence interval
NASH: Non-alcoholic steatohepatitis
MRI: Magnetic resonance imaging
US: Ultrasound
CT: Computed tomography
ML: Machine learning
DL: Deep learning
RF: Radio frequency
PDFF: Proton density fat fraction
PRISMA: Preferred Reporting Items for Systematic Review and Meta-Analysis
TP: True positive
TN: True negative
FP: False positive
FN: False negative
SROC: Summary receiver operating characteristic curves
AUC: Area under the curve
