# Supplementary material for: Image-based AI diagnostic performance for fatty liver: a systematic review and meta-analysis
Source: BMC Med Imaging. 2023 Dec 11;23:208. doi: 10.1186/s12880-023-01172-6 (PMC10712108; doi:10.1186/s12880-023-01172-6)

Supplementary materials

Table S1. Search strategies

| **Database** | **Search strategy** | **Results** | |
| --- | --- | --- | --- |
| 1)PubMed  (To December 24,2022 ) | #1 | “Algorithms”[Mesh] OR "Artificial Intelligence"[Mesh] OR "Machine Learning"[MeSH] OR "Deep Learning"[Mesh] OR "Support Vector Machine"[Mesh] OR “Regression Analysis”[Mesh] OR "Least-Squares Analysis"[Mesh] OR "Decision trees"[MeSH] OR "Random Forest"[Mesh] OR "Logistic Models"[Mesh] OR "Neural Networks, Computer"[Mesh] OR algorithm*[tiab] OR AI[tiab] OR computational intelligence[tiab] OR machine intelligence[tiab] OR Hierarchical Learning*[tiab] OR Support Vector Network[tiab] OR SVM[tiab] OR Logit Model*[tiab] OR least squares[tiab] OR stepwise regression[tiab] OR Neural Network Model*[tiab] OR Perceptron*[tiab] OR Connectionist Model*[tiab] OR k-nearest neighbour[tiab] OR bayesian[tiab] OR naïve bayes[tiab] OR Xgboost[tiab] OR adaboost[tiab] OR gradient boosting machine*[tiab] | 1176702 |
|  | #2 | "Non-alcoholic Fatty Liver Disease"[Mesh] OR nonalcoholic fatty liver disease*[tiab] OR fatty liver*[tiab] OR NAFLD[tiab] OR nonalcoholic steatohepatiti*[tiab] OR NASH[tiab] OR steatohepatiti*[tiab] OR liver steatos*[tiab] OR MAFLD[tiab] OR Metabolic-associated fatty liver disease[tiab] OR Metabolic dysfunction-associated fatty liver disease[tiab] | 55101 |
|  | #3 | "Diagnosis, Computer-Assisted"[Mesh] OR "Early Diagnosis"[Mesh] OR "Sensitivity and Specificity"[Mesh] OR "Area Under Curve"[Mesh] OR detect*[tiab] OR identif*[tiab] OR early diagnos*[tiab] OR auc[tiab] OR sensitivity[tiab] OR specificity[tiab] | 7191950 |
|  | #4 | #1 AND #2 AND #3 | 1382 |
| 2) Embase  (To December 24, 2022) | #1 | Algorithms/exp OR 'Artificial Intelligence'/exp OR 'Machine Learning'/exp OR 'Deep Learning'/exp OR 'Support Vector Machine'/exp OR 'Regression Analysis'/exp OR 'Least-Squares Analysis'/exp OR 'Decision trees'/exp OR 'Random Forest'/exp OR 'Logistic Models'/exp OR 'Neural Networks, Computer'/exp OR algorithm*:ab,ti,kw OR AI:ab,ti,kw OR ‘computational intelligence’:ab,ti,kw OR ‘machine intelligence’:ab,ti,kw OR ‘Hierarchical Learning*’:ab,ti,kw OR ‘Support Vector Network’:ab,ti,kw OR SVM:ab,ti,kw OR ‘Logit Model*’:ab,ti,kw OR ‘least squares’:ab,ti,kw OR ‘stepwise regression’:ab,ti,kw OR ‘Neural Network Model*’:ab,ti,kw OR Perceptron*:ab,ti,kw OR ‘Connectionist Model*’:ab,ti,kw OR ‘k-nearest neighbour’:ab,ti,kw OR bayesian:ab,ti,kw OR ‘naïve bayes’:ab,ti,kw OR Xgboost:ab,ti,kw OR ‘support vector machine’:ab,ti,kw OR SVM:ab,ti,kw OR Xgboost:ab,ti,kw OR adaboost:ab,ti,kw OR ‘gradient boosting machine*’:ab,ti,kw | 1618437 |
|  | #2 | 'Non-alcoholic Fatty Liver Disease'/exp OR ‘nonalcoholic fatty liver disease*’:ab,ti,kw OR ‘fatty liver*’:ab,ti,kw OR NAFLD:ab,ti,kw OR ‘nonalcoholic steatohepatiti*’:ab,ti,kw OR NASH:ab,ti,kw OR steatohepatiti*:ab,ti,kw OR ‘liver steatos*’:ab,ti,kw OR MAFLD:ab,ti,kw OR ‘Metabolic-associated fatty liver disease’:ab,ti,kw OR ‘Metabolic dysfunction-associated fatty liver disease’:ab,ti,kw | 97261 |
|  | #3 | 'Diagnosis, Computer-Assisted'/exp OR 'Early Diagnosis'/exp OR 'Sensitivity and Specificity'/exp OR "Area Under Curve"/exp OR detect*:ab,ti,kw OR identif*:ab,ti,kw OR ‘early diagnos*’:ab,ti,kw OR auc:ab,ti,kw OR sensitivity:ab,ti,kw OR specificity:ab,ti,kw | 10146779 |
|  | #4 | #1 AND #2 AND #3 | 3440 |
|  | #5 | #4 AND 'conference abstract'/it | 1814 |
|  | #6 | #4 NOT 'conference abstract'/it | 1626 |
| 3)Cochrane Library  (To December 24, 2022) | #1 | Algorithms OR 'Artificial Intelligence' OR 'Machine Learning' OR 'Deep Learning' OR 'Support Vector Machine' OR 'Regression Analysis' OR 'Least-Squares Analysis' OR 'Decision trees' OR 'Random Forest' OR 'Logistic Models' OR 'Neural Networks, Computer' OR algorithm* OR AI OR ‘computational intelligence’ OR ‘machine intelligence’ OR ‘Hierarchical Learning*’ OR ‘Support Vector Network’ OR SVM OR ‘Logit Model*’ OR ‘least squares’ OR ‘stepwise regression’ OR ‘Neural Network Model*’ OR Perceptron* OR ‘Connectionist Model*’ OR ‘k-nearest neighbour’ OR bayesian OR ‘naïve bayes’ OR Xgboost OR ‘support vector machine’ OR SVM OR Xgboost OR adaboost OR ‘gradient boosting machine*’:ab,ti,kw | 103249 |
|  | #2 | 'Non-alcoholic Fatty Liver Disease' OR ‘nonalcoholic fatty liver disease*’ OR ‘fatty liver*’ OR NAFLD OR ‘nonalcoholic steatohepatiti*’ OR NASH OR steatohepatiti* OR ‘liver steatos*’ OR MAFLD OR ‘Metabolic associated fatty liver disease’ OR ‘Metabolic dysfunction associated fatty liver disease’:ab,ti,kw | 8089 |
|  | #3 | 'Diagnosis, Computer-Assisted' OR 'Early Diagnosis' OR 'Sensitivity and Specificity' OR "Area Under Curve" OR detect* OR identif* OR ‘early diagnos*’ OR auc OR sensitivity OR specificity:ab,ti,kw | 331069 |
|  | #4 | #1 AND #2 AND #3 | 565 |
|  | #5 | Select “trails” | 243 |

**Table S2. Description of QUADAS-AI**

| **Domain** | **Signalling question** | **Concerns regarding “risk of bias”** |
| --- | --- | --- |
| **Subject selection** | Accurately characterize the source, size and quality of input data alongside clear patient eligibility criteria? | Risk of bias is judged as “low”, “high”, or “unclear”.  1. If all signalling questions for a domain are answered “yes” then risk of bias can be judged “low”.  2. If any signalling question is answered “no” this flags the potential for bias. Review authors then need to have in-depth discussions to judge risk of bias.  3. The “unclear” category should be used only when insufficient data are reported to permit a judgment. |
|  | Use non-open source data? |  |
|  | Present the rationale and breakdown of its training, validation and test sets? |  |
|  | Perform image pre-processing? |  |
|  | Provide the scanner model information used to acquire imaging data? |  |
| **Index test (AI)** | Performed adequate external evaluation ? |  |
| **Reference standard** | Was the reference standard likely to correctly classify the target condition? |  |
| **Work-flow** | Was the time between the index test and the reference standard reasonable? |  |

**Table S3. Performance of models from 15 included studies.**

| **Author** | **Year** | **TP** | **FP** | **FN** | **TN** | **SE[95%CI]** | **SP[95%CI]** |
| --- | --- | --- | --- | --- | --- | --- | --- |
| G. Li et al | 2008 | 66 | 4 | 2 | 21 | 0.97 [0.90, 1.00] | 0.84 [0.64, 0.95] |
| M. Hájek et al | 2011 | 37 | 7 | 3 | 30 | 0.93 [0.80, 0.98] | 0.81 [0.65, 0.92] |
| U. R. Acharya et al | 2012 | 13 | 0 | 2 | 10 | 0.87 [0.60, 0.98] | 1.00 [0.69, 1.00] |
| F. U. A. A. Minhas et al | 2012 | 28 | 1 | 2 | 38 | 0.93 [0.78, 0.99] | 0.97 [0.87, 1.00] |
| R. Ribeiro et al | 2012 | 27 | 0 | 8 | 40 | 0.77 [0.60, 0.90] | 1.00 [0.91, 1.00] |
| R. T. Ribeiro et al | 2014 | 28 | 0 | 8 | 38 | 0.78 [0.61, 0.90] | 1.00 [0.91, 1.00] |
| M. Owjimehr et al | 2015 | 30 | 2 | 0 | 37 | 1.00 [0.88, 1.00] | 0.95 [0.83, 0.99] |
| L. Saba et al | 2016 | 51 | 2 | 1 | 70 | 0.98 [0.90, 1.00] | 0.97 [0.90, 1.00] |
| V. Kuppili et al | 2017 | 32 | 4 | 4 | 23 | 0.89 [0.74, 0.97] | 0.85 [0.66, 0.96] |
| M. Biswas et al | 2018 | 36 | 0 | 0 | 27 | 1.00 [0.90, 1.00] | 1.00 [0.87, 1.00] |
| V. Sharma et al | 2018 | 44 | 3 | 1 | 42 | 0.98 [0.88, 1.00] | 0.93 [0.82, 0.99] |
| A. Han et al | 2020 | 68 | 2 | 2 | 30 | 0.97 [0.90, 1.00] | 0.94 [0.79, 0.99] |
| E. C. Constantinescu et al | 2021 | 295 | 6 | 37 | 291 | 0.89 [0.85, 0.92] | 0.98 [0.96, 0.99] |
| M. Byra et al | 2022 | 94 | 2 | 15 | 24 | 0.86 [0.78, 0.92] | 0.92 [0.75, 0.99] |
| F. Destrempes et al | 2022 | 33 | 6 | 4 | 23 | 0.89 [0.75, 0.97] | 0.79 [0.60, 0.92] |

**Abbreviations:** TP: true-positive; FP: false-positive; FN: false-negative; TN: true-negative; SE: sensitivity; SP: specificity

**Table S4.** **Pooled effects and Heterogeneity in subgroup analysis.**

|  | **No. of studies** | **SE** | ***P* value** | **I**^2^ | **SP** | ***P* value** | **I**^2^ | **PLR** | ***P* value** | **I**^2^ | **NLR** | ***P* value** | **I**^2^ | **DOR** | ***P* value** | **I**^2^ | **SROC** |
| --- | --- | --- | --- | --- | --- | --- | --- | --- | --- | --- | --- | --- | --- | --- | --- | --- | --- |
| **Overall** | 15 | 0.92 | 0.001 | 60.1 | 0.94 | <0.001 | 63.8 | 12.67 | <0.001 | 65.8 | 0.09 | 0.049 | 41.0 | 182.36 | 0.022 | 47.2 | 0.98 |
| **Algorithm** |  |  |  |  |  |  |  |  |  |  |  |  |  |  |  |  |  |
| Conventional Machine learning | 9 | 0.94 | 0.143 | 34.3 | 0.91 | 0.058 | 46.9 | 8.88 | 0.107 | 39.2 | 0.09 | 0.262 | 20.3 | 128.85 | 0.239 | 22.9 | 0.97 |
| Deep learning | 6 | 0.91 | 0.001 | 75.2 | 0.97 | 0.040 | 57.2 | 19.00 | 0.017 | 63.6 | 0.09 | 0.027 | 60.4 | 272.87 | 0.022 | 62.1 | 0.98 |
| **Region** |  |  |  |  |  |  |  |  |  |  |  |  |  |  |  |  |  |
| Asia | 6 | 0.96 | 0.043 | 56.3 | 0.92 | 0.080 | 49.2 | 9.53 | 0.365 | 8.0 | 0.06 | 0.059 | 53.0 | 222.52 | 0.161 | 36.8 | 0.98 |
| Europe and America | 9 | 0.90 | 0.059 | 46.8 | 0.95 | <0.001 | 70.9 | 14.06 | <0.001 | 77.5 | 0.10 | 0.170 | 31.0 | 170.20 | 0.017 | 57.1 | 0.97 |
| **Transfer-learning** |  |  |  |  |  |  |  |  |  |  |  |  |  |  |  |  |  |
| Yes | 2 | 0.88 | 0.469 | 0.0 | 0.98 | 0.141 | 53.9 | 24.74 | 0.082 | 66.9 | 0.12 | 0.325 | 0.0 | 193.73 | 0.070 | 69.5 | - |
| No | 13 | 0.95 | 0.049 | 43.1 | 0.92 | 0.014 | 52.4 | 10.40 | 0.033 | 46.5 | 0.07 | 0.092 | 36.3 | 181.24 | 0.043 | 44.2 | 0.98 |
| **Reference** |  |  |  |  |  |  |  |  |  |  |  |  |  |  |  |  |  |
| US | 7 | 0.92 | 0.0122 | 63.2 | 0.97 | 0.429 | 0.0 | 23.70 | 0.412 | 1.7 | 0.07 | 0.112 | 41.9 | 416.50 | 0.798 | 0.0 | 0.99 |
| Pathology | 5 | 0.91 | 0.0832 | 51.4 | 0.88 | 0.013 | 68.3 | 6.57 | 0.119 | 45.5 | 0.12 | 0.485 | 0.0 | 64.82 | 0.201 | 33.1 | 0.95 |
| MRI | 3 | 0.92 | 0.0054 | 80.8 | 0.90 | 0.453 | 0.0 | 8.77 | 0.458 | 0.0 | 0.06 | 0.004 | 81.8 | 162.05 | 0.328 | 10.3 | 0.97 |
| **Imaging technique** |  |  |  |  |  |  |  |  |  |  |  |  |  |  |  |  |  |
| Conventional US | 12 | 0.94 | 0.003 | 61.7 | 0.94 | 0.257 | 19.0 | 12.17 | 0.472 | 0.0 | 0.07 | 0.004 | 60.1 | 221.09 | 0.205 | 24.3 | 0.98 |
| Elastography | 2 | 0.89 | 0.951 | 0.0 | 0.96 | <0.001 | 93.1 | 13.71 | <0.001 | 95.8 | 0.12 | 0.720 | 0.0 | 117.13 | 0.003 | 89.1 | - |
| MRI | 1 | 0.93 | - | - | 0.81 | - | - | 4.89 | - | - | 0.09 | - | - | 54.33 | - | - | - |

Abbreviations: NO: number; SE: sensitivity; SP: specificity; PLR: positive likelihood ratio; NLR: negative likelihood ratio; DOR: diagnostic odds rate; US: ultrasound; MRI: magnetic resonance imaging.

**Figure S1.** Quality assessment of included articles using the QUADAS-AI criteria.


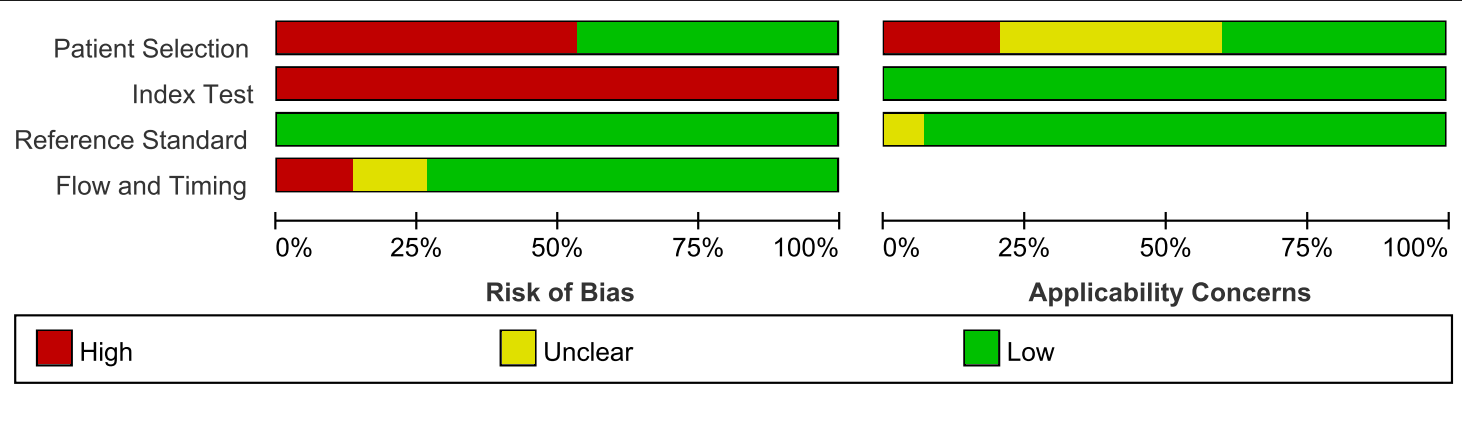


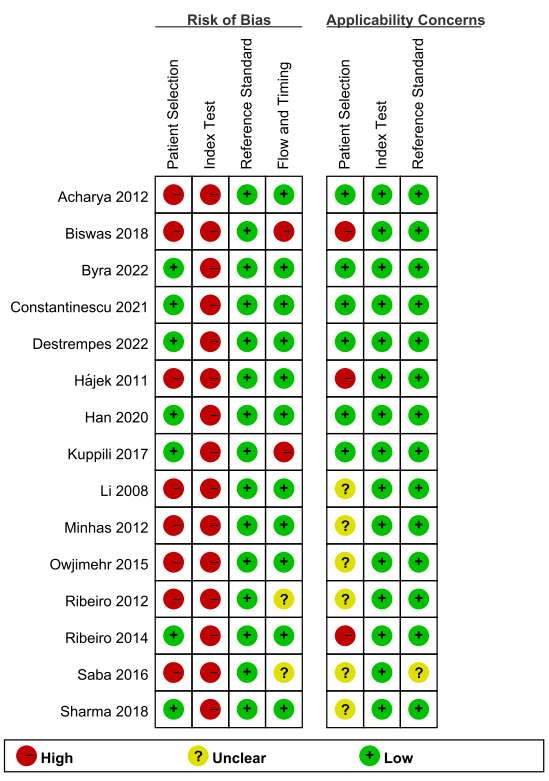

Supplement: Supplementary file 1 — Supplementary Material 1: Table S1. Search strategies; Table S2. Description of QUADAS-AI; Table S3. Performance of models from 15 included studies; Table S4. Pooled effects and Heterogeneity in subgroup analysis; Figure S1. Quality assessment of included articles using the QUADAS-AI criteria. [file 12880_2023_1172_MOESM1_ESM.docx]
